# Structural and compositional mismatch between captive and wild Atlantic salmon (*Salmo salar*) parrs’ gut microbiota highlights the relevance of integrating molecular ecology for management and conservation methods

**DOI:** 10.1111/eva.12658

**Published:** 2018-06-30

**Authors:** Camille Lavoie, Maxime Courcelle, Baptise Redivo, Nicolas Derome

**Affiliations:** ^1^ Biology Department Laval University Quebec QC Canada; ^2^ Institut de Biologie Intégrative et des Systèmes (IBIS) Laval University Quebec QC Canada; ^3^ Institut des Sciences de l’Évolution (ISEM) Montpellier University Montpellier France; ^4^ University of Namur Namur Belgium

**Keywords:** 16S SSU rRNA gene, aquaculture, Atlantic salmon, host–microbiota interactions, metabarcoding sequencing, microbial ecology, stocking

## Abstract

Stocking methods are used in the Province of Quebec to restore *Salmo salar* populations. However, Atlantic salmon stocked juveniles show higher mortality rates than wild ones when introduced into nature. Hatchery environment, which greatly differs from the natural environment, is identified as the main driver of the phenotypic mismatch between captive and wild parrs. The latter is also suspected to impact the gut microbiota composition, which can be associated with essential metabolic functions for their host. We hypothesized that hatchery‐raised parrs potentially recruit gut microbial communities that are different from those recruited in the wild. This study evaluated the impacts of artificial rearing on gut microbiota composition in 0+ parrs meant for stocking in two distinct Canadian rivers: Rimouski and Malbaie (Quebec, Canada). Striking differences between hatchery and wild‐born parrs’ gut microbiota suggest that microbiota could be another factor that could impact their survival in the targeted river, because the microbiome is narrowly related to host physiology. For instance, major commensals belonging to *Enterobacteriaceae* and *Clostridiacea* from wild parrs’ gut microbiota were substituted in captive parrs by lactic acid bacteria from the *Lactobacillaceae* family. Overall, captive parrs host a generalist bacterial community whereas wild parrs’ microbiota is much more specialized. This is the very first study demonstrating extensive impact of captive rearing on intestinal microbiota composition in Atlantic salmon intended for wild population stocking. Our results strongly suggest the need to implement microbial ecology concepts into conservation management of endangered salmon stocks supplemented with hatchery‐reared parrs.

## 1 INTRODUCTION

Demographic decline of Atlantic salmon population in the Province of Quebec led to the introduction of government programs to restore endangered salmon stock in rivers. Most of stocking programs involve the introduction of hatchery‐raised juveniles in rivers, usually 0+ or 1+ parrs (Caron, Fontaine, & Cauchon, 2006; Caron, Fontaine, & Picard, 1999; Milot, Perrier, Papillon, Dodson, & Bernatchez, 2013), because later life stages were observed to have lower reproductive success (Carr, Whoriskey, & O'reilly, 2004). However, several studies show that stocking methods do not meet expected results as hatchery‐born fish exhibit significantly inferior survival rates (from 0.1% to 0.5%) compared to their wild‐born relatives (4%): (Ford, 2002; Kristiansen, Ottera, & Svasand, 2000; Stringwell et al., 2014; Svasand & Kristiansen, 1990), in addition to a lower fitness (Araki, Berejikian, Ford, & Blouin, 2008; Milot et al., 2013). Overall, physiology of stocked salmon is different than that of wild ones (Poole et al., 2003; Stringwell et al., 2014), but it is still unclear how the physiological mismatch takes place between hatchery‐ and wild‐born individuals. Several factors may contribute to these unsuccessful results, such as a strong local adaptation of wild‐born salmon to natural conditions (Dionne, Caron, Dodson, & Bernatchez, 2008; Perrier, Bourret, Kent, & Bernatchez, 2013), even though juveniles are generated with wild breeders captured from the targeted river in order to provide hatchery‐born juveniles with genetic adaptations from the targeted wild population. Despite a genetic composition issued from the wild population, phenotypic mismatch remains in stocked parrs. The latter is therefore suspected to result from acclimation to hatchery conditions themselves (Milot et al., 2013; Orlov, Gerasimov, & Lapshin, 2006; Stringwell et al., 2014). Indeed, these essentially differ from natural environment in terms of water conditions, food and disease management, all of which strongly influencing microbial environment (Donaldson, Lee, & Mazmanian, 2016; Landeira‐Dabarca, Sieiro, & Alvarez, 2013; Ringo & Olsen, 1999; Vrieze et al., 2014). Besides environmental and developmental conditions such as stress (Boutin, Bernatchez, Audet, & Derome, 2013), life stage cycle (Llewellyn et al., 2015; Stephens et al., 2016; Yan et al., 2016), antibiotic administration (Vrieze et al., 2014) and nutrition (Desai et al., 2012; Gajardo et al., 2016; Landeira‐Dabarca et al., 2013; Reveco, Overland, Romarheim, & Mydland, 2014; Ringo & Olsen, 1999), individual (Boutin, Sauvage, Bernatchez, Audet, & Derome, 2014) and population genotype (Dionne, Miller, Dodson, Caron, & Bernatchez, 2007; Dionne et al., 2008) have also been identified as factors contributing to the recruitment of symbionts housing the gut microbiota. As genetic structure of natural population showed evidence of local adaptation for Atlantic salmon (Dionne et al., 2008; Garcia de Leaniz et al., 2007), it is expected that the genetically controlled gut microbiota is also involved in local adaptation (Dionne et al., 2007). Indeed, *Salmo salar* gut microbiota is specific to its local environment at the first life cycle stages and changes as soon smolts migrate from fresh to saltwater (Llewellyn et al., 2015), suggesting that intestinal bacterial communities would also differ depending on the rearing environment.

Over the last decade, several studies highlighted the substantial benefits that the microbiome confers to its host (Bäckhed, Ley, Sonnenburg, Peterson, & Gordon, 2005; Balcazar et al., 2007; Chabrillon et al., 2005; Gaboriau‐Routhiau et al., 2009; Rawls, Samuel, & Gordon, 2004; Scanlan et al., 2008; Stappenbeck, Hooper, & Gordon, 2002; Sylvain & Derome, 2017; Tremaroli & Bäckhed, 2012). Microbial communities play a key role in fish development as they provide bacterial commensals that will colonize all fish body surfaces: skin, gills, and more importantly the intestinal tract (Boutin et al., 2014; Sylvain & Derome, 2017). Specific bacteria composing the gut microbiota are involved in nutrients degradation (Tremaroli & Bäckhed, 2012), activation of immune cells such as lymphoblasts (Gaboriau‐Routhiau et al., 2009), intestinal epithelium cell renewal (Rawls et al., 2004), and angiogenesis of the intestinal tract (Stappenbeck et al., 2002). In teleosts, epithelium and gut microorganisms actively prevent opportunistic pathogens growth by both acting as a physical barrier (Balcazar et al., 2007; Chabrillon et al., 2005) and promoting antimicrobial molecules synthesis (e.g., bacteriocin, enterocin) (Chanos & Mygind, 2016; Satish Kumar et al., 2011), thereby making the gut microbiota a major factor in the development and maturation of the digestive tract immune system (Fredborg, Theil, Jensen, & Purup, 2012; Rawls et al., 2004). It has also been observed that gene regulation and hormone secretion of the host are affected by metabolites from bacterial activity acting as signal molecules (Tremaroli & Bäckhed, 2012). Recent studies have also linked the microbiota composition with modification in epigenetic patterns in newborns, increasing the need to further investigate the role of the microbiota in the understanding of the phenotypic plasticity in teleosts (Bhat & Kapila, 2017; Cortese, Lu, Yu, Ruden, & Claud, 2016; Indrio et al., 2017; Rossi, Amaretti, & Raimondi, 2011). Because host–microbiota interactions are narrowly related to the host physiology (Donaldson et al., 2016; Klaasen et al., 1993; Liu et al., 2012; Scanlan et al., 2008; Wu & Lewis, 2013; Zhang, Lun, & Tsui, 2015), it is suspected that bacterial composition of microbial communities will tightly adapt to artificial rearing conditions (water composition, food, environmental bacterial community), which in turn will affect the ability of hatchery‐reared parrs to adapt to natural conditions once released.

To determine the impact of artificial rearing on the gut microbiota composition of parrs meant for stocking, we sampled parrs juveniles from two different populations (Malbaie and Rimouski river) belonging to two different designable units (DU) of *Salmo salar*. DU are characterized by “an evidence of discreteness, such as in morphology, life history, behavior and/or neutral genetic markers as well as large disjunctions between populations and occupation of different eco‐geographic regions” (COSEWIC, 2010). The two populations are subjected to conservation stocking, for which juveniles are reared in hatchery until they reach the stage of 0+ parrs before being released into the wild. Captive 0+ parrs, issued from wild breeders and reared in Tadoussac Hatchery (Quebec, Canada), have been sampled and compared to their wild relatives. By hypothesizing that parrs’ gut microbiota composition is influenced more by rearing environment than breeder's genotype, this study aimed to (a) characterize environmental microbiota from hatchery and river waters, (b) characterize the gut microbiota composition from captive and wild‐born parrs from the same genetic population (i.e., Rimouski or Malbaie), and (c) identify symbionts that are specific either to hatchery‐ or wild‐born parrs’ gut microbiota. Three predictions were made as follows: (a) Environmental microbial communities would significantly differ between the two rivers and the hatchery water, (b) gut microbiota composition of captive and wild‐born parrs from the same genetic population would significantly differ, and (c) exclusive taxa would be found in both hatchery‐ and wild‐born parrs’ gut microbiota.

Using 16S SSU rRNA gene metabarcoding, we have been able to determine the microbiota composition of 27 parrs gut samples and water samples from each environment. Our results revealed significant differences between environment and gut microbiota from parrs depending on their origin. Furthermore, diet may be the most important factor contributing to the formation of the parrs’ gut microbiota composition. Overall, this study suggests that environment may overpass genotype for the commensals recruitment: Parrs from the same genetic population, reared in two distinct environments, host a significantly different gut microbiome in terms of structure, diversity, and taxonomic composition. For instance, captive parrs’ microbiota hosts mainly *Lactobacillaceae* whereas their wild relatives host mainly *Enterobacteriaceae*. Consequently, stocked parrs’ gut microbiota may not confer the same metabolic functions as their wild relatives. Although further investigations are needed to understand how the divergence of the gut microbiota between the reared parrs’ microbiota and their wild relatives will affect their survival in the wild, it is now clear that host–microbiome interactions must not be neglected for stocking programs.

## 2 MATERIALS AND METHODS

### 2.1 Samples collection and preparation

Captive parrs were sampled from the Tadoussac hatchery (Quebec, Canada), where salmonids from Malbaie and Rimouski rivers are reared for stocking. According to their time of capture, wild breeders from Malbaie and Rimouski rivers were kept in captivity for a period ranging from 2 months to 5 years before spawning. For both groups, artificial spawning occurred between November 28 and December 12, 2012, and hatching between March 29 and April 9, 2013. During incubation, water temperature was set according to the “modified natural thermal regime” to mimic the natural growth rate of wild salmon juveniles. During their growth, reared juveniles were fed with NutraST (Skretting). Captive parrs from both groups were sampled by August 8, 2013, right before stocking. Wild parrs were collected by electrofishing during the summer of 2013 in the Rimouski River (August 19; 48°21′84.1″N, −68°53′77.79″W) and in the Malbaie River (August 25; 47°77′98.8″N, 70°37′38.2″W). A total of 27 parrs were sampled: five wild parrs from the Rimouski river, six wild parrs from the Malbaie river, eight captive parrs from the Rimouski population, and eight captive parrs from the Malbaie population. Parrs were euthanized with MS‐222 and aseptically dissected to remove mid‐ and distal intestinal content. To determine environmental bacterial community composition, two liters of water from each study site were sampled 1 meter below the surface in sterilized Nalgene bottles. Water was then filtered, using a 3.0‐μm nitrocellulose membrane to exclude both organic matter and eukaryotic cells, and a 0.22‐μm sterile membrane to collect bacteria with peristaltic filtration equipment (Cole Parmer, ThermoFisher Scientific). Materials for filtration such as tubes and filter holders were cleaned with 5% HCl and rinsed with Milli‐Q and sample water before each filtration.

Total gut DNA extraction was undertaken using “QIAmp DNA Stool Kit” (Qiagen). For water samples DNA extraction, “DNeasy Blood & Tissue Kit” (Qiagen) was used on the 0.22‐μm nitrocellulose membrane (i.e., containing bacterial cells). Extractions were made according to each kit instruction manual. A first PCR amplification (PCR1) was performed on beforehand diluted 1:10 DNA samples in sterile water samples. A portion of a universal microbial marker, 16S SSU rRNA gene, was amplified using the 803r‐Brian (5′‐GTG ACT GGA GTT CAG ACG TGT GCT CTT CCG ATC TCT ACC RGG GTA TCT AAT CC‐3′) and 347f‐Brian (5′‐ACA CTC TTT CCC TAC ACG ACG CTC TTC CGA TCT GGA GGC AGC AGT RRG GAA T‐3′) primers. These primers bond on hyperconserved regions of the 16S gene, surrounding the hypervariable V3‐V4 region. PCR amplicons were visualized by electrophoresis on agarose gels (2%_m/v_, SB buffer, 100 V) and purified using AMPure XP beads (Beckman & Coulter) to remove the PCR reagents, including primer dimers. DNA quality of amplicons was determined by spectrophotometry (NanoDrop2000, ThermoFisher Scientific). When needed, samples were diluted to obtain 5–10 ng/μl of DNA. A second PCR amplification (PCR2, barcoding step) was performed using a two markers combination as primers to identify the samples. Another validation was performed after the second amplification by electrophoresis and final purification was completed using AMPure XP beads (Beckman & Coulter). Reagents and PCR settings are described in Supporting Information. Once prepared, samples were sent to the sequencing facility of the Institut de Biologie Integrative et des Systèmes (IBIS) of Laval University, Quebec, Canada, for paired‐end sequencing under the MiSeq Illumina platform, using a read length of 2 × 300 pb and V3 kit reagent.

### 2.2 Sequence analysis and statistics

The assembly of paired‐end sequences was performed using QIIME (v.1.9.1) (Edgar, 2010) and PANDAseq (v.1.0) (Maselle, Bartram, Truszkowski, Brown, & Neufeld, 2012). Only paired sequences between 400 and 500 pb with a minimum overlap of 100 bp were kept for further analysis. Chimeric sequences were then removed using QIIME (Usearch61) to ensure that assembled sequences truly resulted from the same operational taxonomic unit (OTU). OTUs from each sample were assigned to the paired reads using the de_novo method at 97% similarity with the SILVA123 database. An OTU table was then obtained and processed using R (3.2.3) and Phyloseq package. A total of 241,016 OTUs was obtained. Before data analysis, OTUs were filtered to remove *Unidentified* taxa. This step reduced the OTUs number to 212,352. All taxa under 0.005% of relative abundance were then removed (Bokulich et al., 2013). The remaining 1,067 OTUs were used for subsequent analysis.

Structure and composition of parrs’ gut microbiota were investigated as follows: Alpha‐diversity was calculated using the Shannon index; richness and evenness alone were estimated with Chao1 and Pielou's indexes, respectively. A nonparametric variance analysis (Kruskal–Wallis) and Kruskal–Wallis post hoc tests were performed on Shannon index to determine whether alpha‐diversity of the gut microbiota from each group was similar. For network analysis, Spearman correlation was calculated between each gut sample. Significant correlations (<5% with Bonferroni correction) with a minimum coefficient of 0.3 were kept for the creation of an interaction network under Cytoscape (v.3.5.1). Gut microbiota composition was also compared between each site using a permutation‐based multivariate analysis of variance (PERMANOVA) of UniFrac distances (weighted and unweighted). UniFrac distances use phylogenetic information of the sequences, comparing the similarity of sequences between samples. A principal coordinate analysis (PCoA) was performed on both weighted and unweighted UniFrac distances, allowing the visualization of individual and group differences of the gut microbiota composition. An analysis of the multivariate homogeneity of group dispersion (variances) was performed on the weighted UniFrac distances using the betadisper function in vegan package. This test allowed us to visualize the dispersion of the microbiota taxonomic structure within captive and wild parrs’ samples. Identification of main OTUs composing the water and gut microbiota was performed by representing the twenty most abundant OTUs of each environment. OTUs were grouped according to family taxonomic rank and visualized with abundance barplots, using ggplot2 package under R. An additional abundance barplot was generated to allow the visualization of the gut and water microbiota composition according to the phylum taxonomic rank. At last, further analysis of gut microbiota composition was carried out by comparing the top 5 OTUs composing each parrs’ group microbiota, represented at the genus level.

## 3 RESULTS

A total of 4,108,663 sequences were obtained after the sequencing and the assembly of paired sequences that were distributed within 23 phyla and assigned to 742 bacterial genera. After filtration (OUT relative abundance threshold of 0.005%), 1,067 OTUs were kept for the analysis of the water and gut microbiota.

### 3.1 Microbiota structure analysis

**Figure 1:**
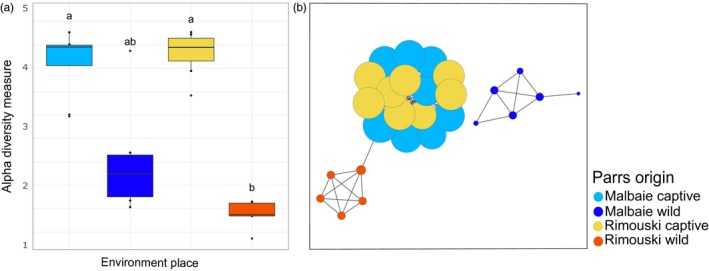
(a) Alpha‐diversity of gut microbiota is lower in wild parrs when compared to their captive relatives. Shannon diversity indexes of gut microbiota are represented in boxplot regarding of parrs location. (b) Environmental conditions are the main driver of gut microbiota. Composition of the 27 microbiota samples, constructed with Cytoscape v.3.2.1, illustrates co‐related samples based on Spearman coefficient (*r* > 0.3, *p*‐value < 0.01). Each node (dot) represents a gut microbiota sample. The node size is proportional to the number of connections a sample makes with other samples, where captive parrs microbiota shows higher number of connections within its group than wild parrs

Analysis of the alpha‐diversity (Shannon index) of the gut microbiota (Figure 1a) showed that captive‐bred parrs housed a much more diversified microbiota than wild parrs. This is especially true for Rimouski population: Wild parrs’ (RWP) mean Shannon index (1.17) was significantly lower than captive parrs’ (RCP: 4.03, *p*‐value = 0.00089, MCP: 3.91, *p*‐value = 0.00152). Malbaie wild parrs (MWP) (mean Shannon index: 2.12) exhibited an intermediate richness between Malbaie captive‐bred parrs and Rimouski wild parrs. This result is mostly explained by one outlier (MWP18), which presented a much higher diversity of symbionts than its relatives. Alpha‐diversity measurement boxplots for Shannon, Chao1, and Pielou's evenness indexes (for water and gut samples), as well as a table indicating their respective values are provided in supporting information (Supporting Information Figures S1, S2, S3 and Table S3).

Network analysis (Figure 1b) shows that individual gut microbiota from captive‐bred parrs issued from both Rimouski and Malbaie populations are strongly connected, with a mean Spearman coefficient of 0.600 (see Supporting Information Table S4 in supporting information for Spearman correlation values). However, individual gut microbiota from wild‐born parrs from both populations are not connected to each other and, more importantly, are either loosely (RWP) or not (MWP) related at all to their respective captive relatives. Moreover, individuals’ gut microbiota from MWP was not connected to any other group. At last, wild‐born parrs mean Spearman coefficient correlation was higher for RWP (mean coefficient: 0.800) than for MWP samples (mean coefficient: 0.461), suggesting lower microbiota compositional homogeneity in MWP when compared to RWP.

**Figure 2:**
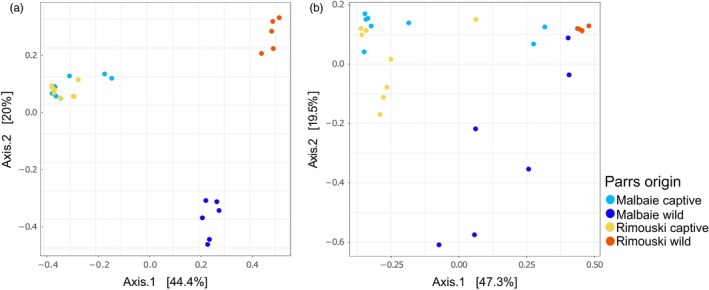
Principal coordinate analysis (PCoA) based on unweighted (a) and weighted (b) UniFrac distances of samples, showing the clustering of samples by their environmental origin. Each dot represents one sample

**Figure 3:**
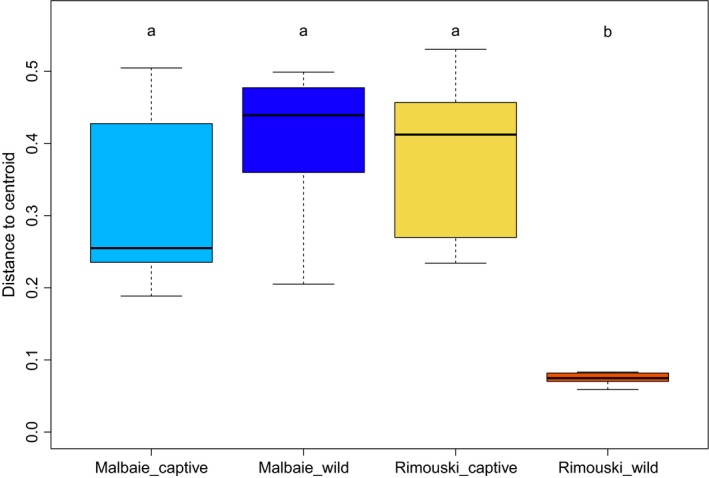
Interindividual variance of the gut microbiota composition is exhibited for each parrs’ group. The boxplot represents the distance to the centroid of the weighted UniFrac distances within each group, based on an analysis of the multivariate homogeneity of dispersion (variances) of samples. The letters represent the ANOVA results of the distance to the centroid for each group

Principal coordinates analysis (PCoA) either based on unweighted (Figure 2a) or weighted (Figure 2b) UniFrac distances showed a clustering of individual gut microbiota according to the origin of the fish: Captive‐bred parrs are grouped, whereas wild parrs’ populations are differentiated from one another and from their captive relatives. Clustering was even more pronounced with unweighted distance matrices (Figure 2a). The PERMANOVA based on weighted (Table 1) and unweighted (Table 2) UniFrac metric distances revealed significant to highly significant differences between most groups, the lowest differentiation being detected for captive‐bred parrs, which was not significant for weighted UniFrac distances (*p*‐value = 0.116). The analysis of the multivariate homogeneity of group dispersion (variances) revealed a higher interindividual variation for captive parrs and MWP group when compared to RWP (Figure 3). No significant difference was obtained when comparing MCP interindividual variances to MWP (*p‐*value = 0.4271981). However, it is possible to assess the higher interindividual variation for MWP group by the outlier MWP18, which shows a more diverse gut microbiota composition than its relatives from the same origin as well as a singular bacterial composition.

**Table 1:** PERMANOVA results of the weighted UnifFrac distances, comparing microbiota composition of each parrs’ group, showing significant differences between most groups

Weighted UniFrac			
	MCP	RCP	MWP
RCP	0.116***		
MWP	0.003**		
RWP		0.002**	0.021*

Notes

MCP: Malbaie captive parrs; MWP: Malbaie wild parrs; RCP: Rimouski captive parrs; RWP: Rimouski wild parrs.

Signif. Codes: 0 “***” 1 “**” 0.01 “*” 0.05.

**Table 2:** PERMANOVA results of the unweighted UnifFrac distances, comparing microbiota composition of each parrs’ group, showing significant differences between most group

Unweighted UniFrac			
	MCP	RCP	MWP
RCP	0.001***		
MWP	0.001***		
RWP		0.005**	0.004**

Notes

MCP: Malbaie captive parrs; MWP: Malbaie wild parrs; RCP: Rimouski captive parrs; RWP: Rimouski wild parrs.

Signif. Codes: 0 “***” 0.001 “**” 0.01.

### 3.2 Environment microbiota composition

**Figure 4:**
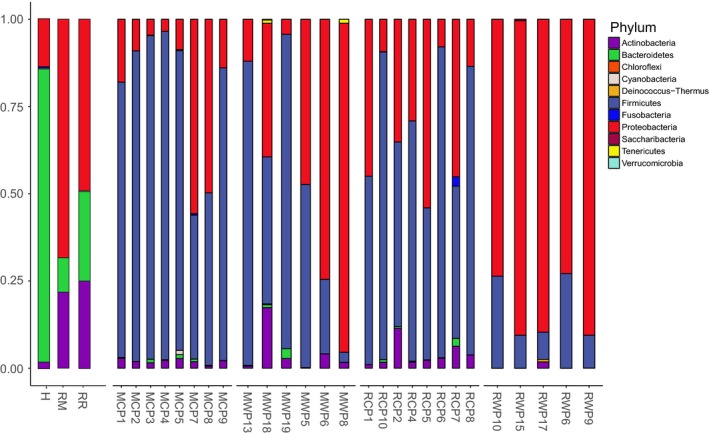
Total environment and gut microbiota composition for every samples represented at the phylum level. H, Hatchery water; MR, Malbaie River; RR, Rimouski River; MCP, Malbaie captive parrs; MWP, Malbaie wild parrs;RCP, Rimouski captive parrs; RWP, Rimouski wild parrs

**Figure 5:**
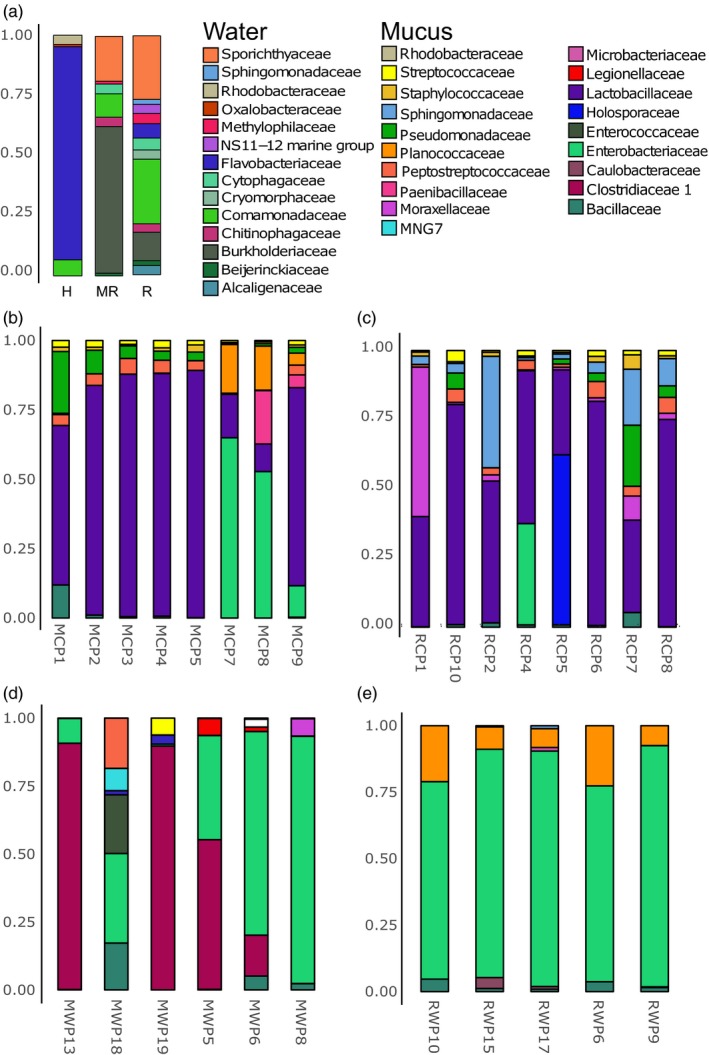
Environment and gut microbiota composition of the 20 most abundant OTUs found in every samples, represented at the family level. (a) Environment water; H, Hatchery; MR, Malbaie River; RR, Rimouski River; (b) Malbaie captive parrs; (c) Rimouski captive parrs; (d) Malbaie wild parrs; (e) Rimouski wild parrs

The analysis of the water bacterial community composition at the phylum level revealed the presence of *Bacteroidetes*,* Proteobacteria,* and *Actinobacteria* in every environment (Figure 4), but respective abundance levels varied between sampling sites, particularly for *Actinobacteria* which was less abundant in hatchery water. The bacterial taxonomic composition, characterized at the family level, however, showed diagnostic taxa for every single microbial niche: environmental water (hatchery and both rivers) and fish gut microbiota composition (Figure 5). For water samples, the 20 most abundant OTUs from each site accounted for an average of 64% of the total bacterial composition. For hatchery water, *Flavobacteriaceae* (63.6%) was the main taxa composing the water bacterial community (i.e., bacterioplankton). In contrast, Malbaie and Rimouski rivers exhibited a more diversified bacterial community, dominated with *Sporichthyaceae* and *Burkholderiaceae*, composing, respectively, 13.6% and 44.3% of the Malbaie river bacterioplankton, and composing, respectively, 13.8% and 6.20% for Rimouski river bacterioplankton.

### 3.3 Gut microbiota composition

**Figure 6:**
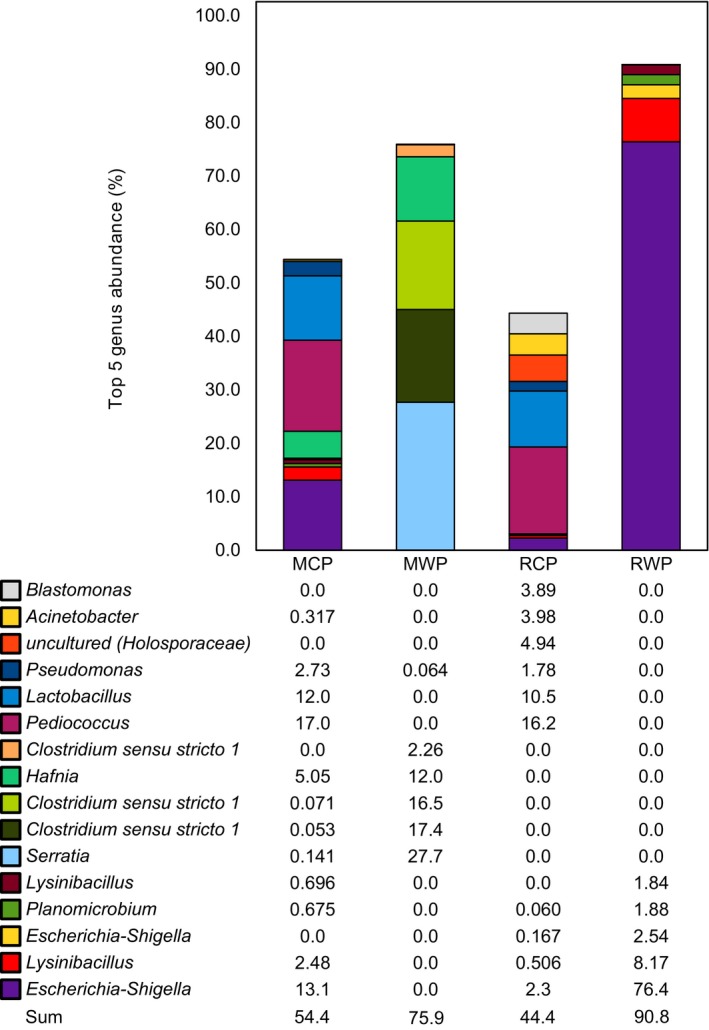
Comparison of the five most abundant OTUS for every parrs’ group represented at the Genus level shows defined profile depending of parrs origin; Captive parrs microbiota is characterized by the presence of the same OTU from the *Lactobacillus* and *Pediococcus* genus. The top 5 OTUs from wild parrs are distinct, and no OTU from the top 5 is shared between the two groups. Relative abundance in % of the top 5 OTUs is compared between each group in the table below

At the phylum level, the gut microbiota composition revealed the presence of *Proteobacteria*,* Firmicutes* and *Actinobacteria* in each group (Figure 4). However, a much more heterogeneous composition was highlighted within groups when the bacterial composition was analyzed at the family level (Figure 5). For each parrs’ population, the 20 most abundant OTUs in gut (Figure 5b,c,d,e), identified at the family level, accounted for 68.3% in MCP, 64.7% in RCP, 90.0% in MWP and 97.5% in RWP. Moreover, diversity index was higher for captive parrs when compared to wild parrs (Figure 1a). Taken together, these results show that dominant OTUs were less abundant in captive parrs than in their wild relatives (see relative abundance of the twenty most abundant OTUs in Appendix 1). In addition, it is possible to identify commensal bacteria that were either specific to the hatchery or to the water by observing the 20 most abundant OTUs. For hatchery‐born parrs (Figure 5b,c), *Lactobacillaceae* is the main taxa found in every captive sample at high levels, with a mean abundance of 40.7% for MCP and 36.4% for RCP. For RCP group, *Sphingomonadaceae* (6.12%), *Moraxellaceae* (7.38%), and *Holosporaceae* (4.94%) were identified as the three major taxa after *Lactobacillaceae*. Both RCP and MCP showed one (RCP) or three (MCP) samples containing *Enterobacteriaceae* in their microbiota, with a respective mean abundance of 2.28% and 13.1%. MCP group, in addition to *Lactobacillaceae* and *Enterobacteriaceae,* was characterized by the presence of *Planococcaceae* (3.85%), *Pseudomonadaceae* (3.47%), and *Bacillaceae* (1.10%). RWP parrs composed by far the most homogenous and structured group, showing a gut microbiome overdominated by *Enterobacteriaceae* (80.2%), followed by *Planococcaceae* (13.0%) and *Bacillaceae* (2.33%). MWP parrs composed a more heterogenous and less structured group. It was possible to identify dominant symbionts such as *Enterobacteriaceae* (35.2%) in all individuals, except for MWP19 and *Clostridiaceae* (37.0%) in most of the individuals (see Appendix 2 for the taxa relative abundances for each sample). Strikingly, sample MWP18 exhibited a very distinct profile characterized by higher microbiota diversity, including *Peptostreptococcaceae*,* Enterococcaceae* and *Enterobacteriaceae, Bacillaceae, MNG7,* and *Bacillaceae*. At last, the two dominant taxa that were shared between captive and wild parrs for both populations belong to *Bacillaceae* and *Enterobacteriaceae*. Then, *Streptococcaceae, Peptostreptococcaceae, Enterobacteriaceae* were found in captive and wild parrs from Malbaie population, and both *Planococcaceae* and *Bacillaceae* were shared by captive and wild parrs from Rimouski (Figure 5). The five most important OTUs, shown in Figure 6 at the genus level, are compared between each parrs’ group with their respective relative abundance. Two OTUs are shared between MCP and RCP: *Pediococcus* and *Lactobacillus*, which represent, respectively, 17.0% and 12.0% for MCP and 16.2% and 10.5% for RCP. The top five OTUs for captive parrs mostly belong to the *Lactobacillaceae, Holosporaceae, Moraxellaceae, Enterobacteriaceae Sphingomonadaceae, Planoccocaceae* and *Pseudomonadacea*e families, as those from wild parrs are mostly members of *Enterobacteriaceae, Clostridiaceae 1, Enterococcaceae* and *Planococcaceae* family but none of the top 5 OTUs were shared between MWP and RWP.

## 4 DISCUSSION

The purpose of this study was to assess the effect of both host population and rearing environment on gut microbiota taxonomic composition of *Salmo salar* parrs intended for river stocking. Hatchery‐reared parrs were generated with breeders issued from two wild populations (i.e., Rimouski and Malbaie rivers), whereas wild parrs were naturally born and grown in their respective rivers. Overall, our results clearly demonstrate that environmental conditions (i.e., hatchery versus river) had the most prevalent effect on gut microbiota taxonomic composition. Substantial impacts on the fish's energetic and behavioral phenotypes resulting from hatchery‐rearing conditions have been documented in numerous studies and are suspected of greatly reduce survival rates of stocked fishes (Milot et al., 2013; Stringwell et al., 2014). It has also been demonstrated that reared fish show a different methylation pattern than that of their wild relatives (Le Luyer et al., 2017). As energetic and behavioral phenotypes are controlled by gut microbiota activity (Tremaroli & Bäckhed, 2012) and the latter is suspected to impact epigenetic patterns (Bhat & Kapila, 2017; Cortese et al., 2016; Indrio et al., 2017; Rossi et al., 2011), our study is of prime interest as it is the first to demonstrate the extensive impact of captive rearing on gut microbiota composition in Atlantic salmon parrs meant for stocking. By identifying significant differences in terms of both structure and taxonomic composition between captive parrs and their wild relatives, the present work evidenced that acclimation to artificial rearing is also observable at the host–microbiota level. This result is striking enough as it is now well established that salmonids’ microbiota composition is regulated by host genotype (Boutin et al., 2014), population genotype (Dionne et al., 2007), life cycle stage (Llewellyn et al., 2015; Stephens et al., 2016; Yan et al., 2016), and environment (Llewellyn et al., 2015). Importantly, our results suggest that acclimation to artificial rearing overpasses host genotype effect on modeling microbiota composition at both individual and population level.

Environment is an important driver for gut microbiota structuration and composition: Compared to wild parrs, hatchery‐reared parrs exhibited a higher bacterial diversity (Figure 1), combined with lower disparity (i.e., most of OTU sharing similar relative abundance), both of which are characteristic of an immature microbial community with low structuration (Burns et al., 2016; Llewellyn et al., 2015; Sylvain & Derome, 2017). Hatchery‐rearing conditions are stable with almost no competition for food, thus being very different to those encountered in the natural environment (McPhee, 2004). As such, the low structuration of hatchery‐reared parrs’ microbiota likely results from relaxed selective pressure (Derome, Duchesne, & Bernatchez, 2006; Fisher, 1930), which would translate into a random recruitment of pioneering bacterial symbionts. The hatchery diet itself would explain such results: Commercial pellets are enriched with nutrients from various origins, including substantial amount of vegetable proteins (e.g., soya) (Feng, Hu, Luo, Zhang, & Chen, 2010; Tanaka, Ootsubo, Sawabe, Ezura, & Tajima, 2004). Enriched food with vegetable proteins and carbohydrates was observed to significantly impact gut microbiota composition in fishes by increasing diversity and richness and more specifically by inducing a significant increase in Firmicutes symbionts, including lactic acid bacteria (LAB), mostly belonging to *Lactobacillaceae, Enterococcaceae,* and *Streptococcaceae* (Desai et al., 2012; Gajardo et al., 2016). In our study, commercial diet impact translated into the systematic overdominance of the *Lactobacillaceae* family in every captive parrs’ gut microbiota, whereas this family was absent from the top 20 OTUs composing the wild parrs’ gut microbiota. Consistently, gut bacteria belonging in the *Lactobacillaceae* family have only been identified in very small amount, if not totally absent, in salmonids coming from the natural environment (Gajardo et al., 2016; Llewellyn et al., 2015). Moreover, plant meal diet (PMD) has been related to the increase in intestinal inflammation and sensibility to various diseases (Krogdahl, Bakke‐Mckellep, Roed, & Baeverfjord, 2000) as well as decreasing nutrient digestion and absorption (Nordrum, Bakke‐McKellep, Krogdahl, & Buddington, 2000). Taken together, our results suggest that captive parrs’ microbiota composition would therefore be qualified as “generalist” when compared to the highly structured and, thus more specialized, wild parrs’ gut microbiota. This more specialized gut microbiota in wild parrs can be attributed to the higher selective pressures occurring in wild rivers, including a more restricted diet, which is mostly composed of insects’ larvae, crustacean, and annelids (Bell, Ghioni, & Sargent, 1994). Consequently, the most important environmental factor for the recruitment of intestinal symbionts in teleosts could be associated with diet. This factor could explain most of the heterogeneity of the gut microbiota, the latter being greatly related by its capacity to assimilate nutrients (Tremaroli & Bäckhed, 2012). Therefore, controlling gut microbial symbionts in hatchery could be of prime interest to secure the recruitment of key adaptive microbiota functions of wild parrs.

Regarding both wild‐born parrs’ populations, gut microbiota composition significantly differed accordingly to their population origin, thus confirming our previous work on wild populations, stating that gut microbiota composition at early life stages is mostly driven by geography (study site) (Llewellyn et al., 2015). Furthermore, network analysis, PCoA, and PERMANOVA evidenced a great contribution of the environment. It is interesting that MWP group showed fewer correlations within samples when compared to RWP, thus indicating more intragroup variations of the gut microbiota composition in MWP population. This result is confirmed by the analysis of the multivariate homogeneity of dispersion (Figure 3). Given that recruitment of specific bacterial symbionts is associated with host‐specific genes in salmonids (Boutin et al., 2014), it would be straightforward to investigate further the genetic structure of the Malbaie river *Salmo salar* population in order to assess whether this population is genetically introgressed with allopatric breeders (i.e., issued from other river populations).

Hatchery‐raised parrs issued from both river populations share a similar richness index and are strongly correlated by their composition itself, as shown with both network analysis and PCoA based on weighted and unweighted UniFrac (Figure 2), which clusters both captive parrs populations in a single, isolated, group. Consistently, PERMANOVA on unweighted UniFrac distances showed the weakest, but significant, differentiation between captive parrs from both genetic origins (i.e., Malbaie versus Rimouski), and no differentiation was found when performing the analysis on weighted UniFrac (Table 1). Therefore, even though samples cluster mostly by environment, thus suggesting this factor overpasses genetic origin in controlling gut microbiota composition, it also suggests that genetic origin is still exerting a minimal control on bacteria recruitment. At last, sanitary management in hatcheries, due to high density, impairs microbial environment and gut microbiota (Carlson, Leonard, Hyde, Petrosino, & Primm, 2017; Nakayama et al., 2017), thus amplifying microbiota divergence between hatchery‐ and wild‐born parrs.

In addition to the phenotypic mismatch between captive and wild salmonids from the same genetic population (Araki et al., 2008; Milot et al., 2013; Poole et al., 2003; Stringwell et al., 2014), hatchery‐raised parrs are also facing an important microbiota mismatch regarding key microbial symbionts recruited by their wild relatives. Overall, it came out that extensive differences observed between hatchery and river environmental microbiota composition suggest that stocked parrs are exposed to a totally different microbial environment when released into the target river. Knowing that their gut microbiota composition differs from that of wild parrs, exposure to a very different environmental microbial community could lead to an impairment of colonization resistance to wild opportunistic pathogens, thus potentially favoring disease. The sudden transfer from hatchery water to river environment exerts a considerable stress, which is expected to trigger a transient dysbiosis (i.e., altered microbiota activity) giving further opportunities for pathogens to infect fish tissues (Bonga, 1997; Boutin et al., 2013; Seghouani, Garcia‐Rangel, Füller, Gauthier, & Derome, 2017). At last, overdominance of *Lactobacillaceae* in captive parrs is expected to generate a reduced capacity to assimilate nutrients from wild preys. Whether hatchery “imprinting” on microbiome is transient or permanent in stocked fishes after release into the targeted river is yet to be investigated. Even though two previous studies on rainbow trout observed that the first diet type had no effect on the microbiota composition after a diet shift (Ingerslev et al., 2014; Michl et al., 2017), several studies have also highlighted the long‐term effects of the microbiome associated with early life stage cycle diet on the host physiology in human (Indrio et al., 2017; Mischke & Plösch, 2013) and rainbow trout (Ingerslev et al., 2014). Furthermore, Rhossart et al. (2017) demonstrated that host fitness‐promoting traits regarding naturally occurring diseases were associated with the “natural microbiome” of wild mice. Indeed, domestic microbiota was associated with greater inflammation and lower resistance to pathogens relatively to wild individual microbiota. Those results are in concordance with other studies reporting that reared fishes fed with a PMD have shown higher level of gut inflammation (Krogdahl et al., 2000; Nordrum et al., 2000), which is now recognized as an important driver of many diseases (Rajani & Jia, 2018). Because the microbiota composition is proven to be actively involved in several metabolism pathways (Tremaroli & Bäckhed, 2012) and immune responses (Rhossart et al., 2017), investigating the relationship between the host energetic phenotype and the microbiome functional repertoire of captive and wild parrs is crucial to assess to what extent the gut microbiota taxonomic mismatch is actually associated to the loss of microbial functions. Evidence of “metabolic imprinting” (Hanley et al., 2010) related to the microbiota composition at early life stage in hatchery could be suspected to contribute to the mitigated impact of supportive breeding programs, in addition to the potential dysbiosis that may occur during stocking. Therefore, further studies are strongly needed to test whether the taxonomic (and functional) microbial mismatch between hatchery‐raised and wild‐born salmons could underlie the lower survival of stocked fishes once released into the wild.

Altogether, these results strongly suggest that the extensive discrepancy between hatchery‐raised and wild parrs’ gut microbiota potentially translates into a phenotypic disadvantage for the former, at least regarding disease resistance and food energetic conversion. Indeed, the two most important symbionts of the wild parrs’ gut microbiota such as *Enterobacteriaceae* (MWP, RWP) and *Clostridiaceae* (MWP only) are at very low levels in most captive parrs (Figure 5) as well as the top 5 OTUs from wild parrs (Figure 6). Therefore, stocked parrs could have developed different metabolic functions from their wild relatives, regardless of their common genetic origin, which could considerably reduce their fitness in natural conditions. It is interesting that in MWP group, one individual (MWP18) harbored a very distinct microbiota composition. Knowing that Malbaie river is subject to stocking, it becomes even more relevant to identify its origin. However, stocked fish from this river are identified by a clipped caudal fin, but MWP18's fin was not mentioned to harbor such a characteristic. Nevertheless, further investigations are needed to test whether such generalist microbiome from captive parrs provides or not key functions ensuring optimal host physiology in the wild.

## 5 CONCLUSION

Given that parrs’ gut microbiota composition is strongly related to the rearing environment, differences in terms of structure and composition between wild and hatchery‐born parrs give valuable information toward improving management of reared fish intended for stocking. Consequently, it becomes even more crucial to investigate the link between environmental and gut microbial communities’ taxonomic composition to get new insights on factors driving differences between captive and wild parrs’ microbiota and therefore their adaptive ability in a given environment. In light of our results, we support the recommendations stated by Milot et al. (2013) to adopt more natural rearing condition and to release juveniles at a younger stage, before the first feeding occurs. As it may be difficult to exactly mimic the natural conditions in hatchery, the implementation of bacterial ecology in supplementation programs could be one possible avenue to investigate looking forward. Unless the hatchery is connected to the targeted river, one avenue would be to provide beneficial bacteria detected in wild populations to hatchery‐reared juveniles through the administration of probiotics. Therefore, further studies are needed to assess how to control the microbiota composition in hatchery and to characterize natural microbiome for each population subjected to supportive breeding programs. To conclude, we strongly believe that implementing host–microbiota evolutionary process and microbial ecology into conservation policies would improve the efficiency of stocking programs for *Salmo salar*, but also for every teleost species suffering a demographic decline.

## DISCLAIMER

Please note that primers used in this work contain Illumina‐specific sequences protected by intellectual property (Oligonucleotide sequences © 2007‐2013 Illumina, Inc). All rights reserved. Derivative works created by Illumina customers are authorized for use with Illumina instruments and products only. All other uses are strictly prohibited.)

## DATA ARCHIVING STATEMENT

Data are available from the Dryad Digital Repository: https://doi.org/10.5061/dryad.5ff8m0q.

## APPENDIX 1 Relative mean abundance (%) of the 20 most abundant OTUs grouped at the family level of the microbiota composition for every group of parrs

### 1.1

**Table:**

	Family	Mean (%)		Family	Mean (%)
RCP	*Bacillaceae*	0.706	RWP	*Bacillaceae*	2.33
	*Enterobacteriaceae*	2.28		*Caulobacteraceae*	1.05
	*Holosporaceae*	4.94		*Enterobacteriaceae*	80.2
	*Lactobacillaceae*	36.4		*Microbacteriaceae*	0.266
	*Moraxellaceae*	7.38		*Planococcaceae*	13.0
	*Peptostreptococcaceae*	2.23		*Sphingomonadaceae*	0.297
	*Pseudomonadaceae*	2.58		Sum	97.1
	*Sphingomonadaceae*	6.13	MWP	*Bacillaceae*	2.66
	*Staphylococcaceae*	0.982		*Clostridiaceae 1*	37.0
	*Streptococcaceae*	1.07		*Enterobacteriaceae*	35.2
	Sum	64.7		*Enterococcaceae*	1.89
MCP	*Bacillaceae*	0.812		*Flavobacteriaceae*	0.611
	*Enterobacteriaceae*	9.71		*Legionellaceae*	1.18
	*Lactobacillaceae*	30.1		*MNG7*	0.726
	*Paenibacillaceae*	1.69		*Moraxellaceae*	0.984
	*Peptostreptococcaceae*	1.53		*nrb16a11*	0.428
	*Planococcaceae*	2.85		*Peptostreptococcaceae*	1.63
	*Pseudomonadaceae*	2.57		*Streptococcaceae*	0.954
	*Staphylococcaceae*	0.532		Sum	83.3
	*Streptococcaceae*	0.807			
	Sum	50.6			

Note

MCP: Malbaie captive parrs; MWP: Malbaie wild parrs; RCP: Rimouski captive parrs; RWP: Rimouski wild parrs.

## APPENDIX 2 Relative abundance (%) of the 20 most abundant OTUs from each group, represented at the family level for every samples

### 2.1

**Table:**

MCP	MCP1	MCP2	MCP3	MCP4	MCP5	MCP7	MCP8	MCP9
*Streptococcaceae*	2.43	2.69	1.95	2.79	1.75	1.26	0.68	1.87
*Staphylococcaceae*	1.81	1.52	0.74	1.51	2.45	0.51	0.70	0.90
*Pseudomonadaceae*	15.19	6.02	3.22	2.35	2.03	0.26	0.96	1.61
*Planococcaceae*	1.56	0.35	0.13	0.19	0.24	17.60	16.61	3.86
*Peptostreptococcaceae*	3.33	3.42	4.88	3.97	3.04	0.10	0.33	3.86
*Paenibacillaceae*	0.31	0.23	0.03	0.42	0.00	0.22	15.58	3.12
*Lactobacillaceae*	53.74	77.18	80.59	80.42	75.15	17.91	11.90	66.45
*Enterobacteriaceae*	0.22	0.11	0.07	0.05	0.00	53.91	45.03	8.51
*Bacillaceae*	10.6	1.90	1.56	1.72	0.93	2.87	2.45	1.15
**RCP**	**RCP1**	**RCP10**	**RCP2**	**RCP4**	**RCP5**	**RCP6**	**RCP7**	**RCP8**
*Streptococcaceae*	0.82	6.37	1.70	1.81	1.10	2.28	2.49	1.96
*Staphylococcaceae*	1.66	0.72	1.19	0.61	0.91	2.26	3.72	1.14
*Sphingomonadaceae*	2.81	2.73	25.61	0.69	1.67	2.58	14.39	7.29
*Pseudomonadaceae*	0.04	3.94	0.00	0.01	1.61	2.05	12.43	3.04
*Peptostreptococcaceae*	1.08	3.99	2.07	2.59	1.14	4.47	2.19	4.55
*Moraxellaceae*	40.77	0.55	2.06	0.22	0.92	1.07	5.30	1.57
*Lactobacillaceae*	45.07	71.28	41.47	50.04	37.11	72.67	28.45	70.22
*Holosporaceae*	0.03	0.00	0.00	0.00	48.65	0.00	0.00	0.00
*Enterobacteriaceae*	0.27	0.06	0.01	25.39	0.05	0.00	0.01	0.03
*Bacillaceae*	1.27	2.18	2.48	1.65	1.13	2.21	4.93	1.64
**MWP**	**MWP13**	**MWP18**	**MCP19**	**MWP5**	**MWP6**	**MWP8**		
*Streptococcaceae*	0.09	0.00	6.56	0.00	0.34	0.19		
*Peptostreptococcaceae*	0.00	9.92	0.00	0.07	0.01	0.00		
*nbr16a11*	0.00	0.00	0.00	0.00	3.84	0.00		
*Moraxellaceae*	0.00	0.00	0.78	0.00	0.00	7.31		
*MNG7*	0.00	4.86	0.00	0.04	0.01	0.00		
*Legionellaceae*	0.00	0.76	0.00	7.37	1.70	0.00		
*Flavobacteriaceae*	0.00	0.80	2.79	0.01	0.00	0.07		
*Enterococcaceae*	0.00	15.94	0.00	0.00	0.00	0.00		
*Enterobacteriaceae*	10.82	18.03	0.68	39.54	72.52	86.17		
*Clostridiaceae 1*	86.46	0.66	80.31	52.19	14.82	0.08		
*Bacillaceae*	0.14	12.09	0.00	0.17	5.62	2.26		
**RWP**	**RWP10**	**RWP15**	**RWP17**	**RWP6**	**RWP9**			
*Sphingomonadaceae*	0.00	0.70	1.03	0.00	0.00			
*Planococcaceae*	21.66	8.32	6.91	23.38	7.72			

Note

MCP: Malbaie captive parrs; MWP: Malbaie wild parrs; RCP: Rimouski captive parrs; RWP: Rimouski wild parrs.
