# Supplementary material for: Structural and compositional mismatch between captive and wild Atlantic salmon (Salmo salar) parrs’ gut microbiota highlights the relevance of integrating molecular ecology for management and conservation methods
Source: Evol Appl. 2018 Jun 30;11(9):1671–85. doi: 10.1111/eva.12658 (PMC6183451; doi:10.1111/eva.12658)
Supplement: Supplementary file 1 [file EVA-11-1671-s001.pdf]

**Supporting informations**

**Tab. 1.** Used reagents for PCR1 and PCR2 and their respective quantity in µL for processing one sample.

| Reagent | Quantity (µL) | |
|---|---|---|
| | PCR 1 | PCR2 |
| Q5 Buffer | 10,0 | 10,0 |
| dNTPs | 1,00 | 1,00 |
| 803r-Br | 2,50 | - |
| 347f-Br | 2,50 | - |
| Forward Primer (predetermined) | - | 1,00 |
| Reverse Primer (markers) | - | 1,00 |
| «High GC enhancer» | 10,0 | 10,0 |
| DNA | 3,00 | 2,00 |
| Q5 Polymerase (New England Biolabs) | 1,00 | 1,00 |
| Sterile water | 20,0 | 24,0 |

**Tab. 2.** DNA amplification conditions for PCR1 and PCR2. The « Denaturation », « Hybridation » and « Elongation » steps were performed 35 times for PCR1 and 12 times for PCR2.

| Step | Temperature (°C) | Duration |
|---|---|---|
| Initial Denaturation | 98.0 | 2 min. |
| Denaturation | 98.0 | 10 sec. |
| Hybridation | 60.0 | 30 sec. |
| Elongation | 72.0 | 30 sec. |
| Final Elongation | 72.0 | 10 min. |

**Tab. 3.** Alpha diversity indexes calculated using Shannon and Chao1 indexes for each environment.

| Alpha diversity indexes | | | |
|---|---|---|---|
| Environment | Shannon | Chao1 | Pielou |
| Hatchery water | 3.56 | 401 | 0.613 |
| Malbaie River | 3.25 | 322 | 0.629 |
| Rimouski River | 4.23 | 430 | 0.646 |
| Malbaie Captive | 3.91 | 572 | 0.582 |
| Rimouski Captive | 4.03 | 109 | 0.369 |
| Malbaie Wild | 2.12 | 583 | 0.609 |
| Rimouski Wild | 1.17 | 69 | 0.219 |

**Tab. 4 :** Spearman correlation values of every samples interactions over 0.3.

| Sample1 | Sample2 | Spearman correlation value |
|---|---|---|
| MCP2 | MCP1 | 0.776727915 |
| MCP3 | MCP1 | 0.736366868 |
| MCP4 | MCP1 | 0.775909662 |
| MCP5 | MCP1 | 0.704556167 |
| MCP7 | MCP1 | 0.431953102 |
| MCP8 | MCP1 | 0.388078451 |
| MCP9 | MCP1 | 0.651246965 |
| RCP1 | MCP1 | 0.657814682 |
| RCP10 | MCP1 | 0.713101864 |
| RCP2 | MCP1 | 0.52373004 |
| RCP4 | MCP1 | 0.587184966 |
| RCP5 | MCP1 | 0.694195151 |
| RCP6 | MCP1 | 0.715943098 |
| RCP7 | MCP1 | 0.545648456 |
| RCP8 | MCP1 | 0.688752949 |
| MCP3 | MCP2 | 0.825522363 |
| MCP4 | MCP2 | 0.849120796 |
| MCP5 | MCP2 | 0.788360953 |
| MCP7 | MCP2 | 0.438159555 |
| MCP8 | MCP2 | 0.379061043 |
| MCP9 | MCP2 | 0.729747891 |
| RCP1 | MCP2 | 0.684093237 |
| RCP10 | MCP2 | 0.774275661 |
| RCP2 | MCP2 | 0.556477547 |
| RCP4 | MCP2 | 0.596498847 |
| RCP5 | MCP2 | 0.73960495 |
| RCP6 | MCP2 | 0.796154201 |
| RCP7 | MCP2 | 0.537454367 |
| RCP8 | MCP2 | 0.748993635 |
| MCP4 | MCP3 | 0.86482209 |
| MCP5 | MCP3 | 0.763691425 |
| MCP7 | MCP3 | 0.425015688 |
| MCP8 | MCP3 | 0.373106271 |
| MCP9 | MCP3 | 0.702676535 |
| RCP1 | MCP3 | 0.726239443 |
| RCP10 | MCP3 | 0.794598341 |
| RCP2 | MCP3 | 0.543984294 |
| RCP4 | MCP3 | 0.600866735 |
| RCP5 | MCP3 | 0.734427035 |
| RCP6 | MCP3 | 0.765117586 |
| RCP7 | MCP3 | 0.495664239 |
| RCP8 | MCP3 | 0.751027405 |
| MCP5 | MCP4 | 0.761723578 |
| MCP7 | MCP4 | 0.436742604 |
| MCP8 | MCP4 | 0.417781413 |
| RCP4 | MCP7 | 0.579445541 |
| RCP5 | MCP7 | 0.381068438 |
| RCP6 | MCP7 | 0.426821709 |
| RCP7 | MCP7 | 0.354970843 |
| RCP8 | MCP7 | 0.353553265 |
| RWP10 | MCP7 | 0.301009506 |
| MCP9 | MCP8 | 0.67005378 |
| RCP1 | MCP8 | 0.321904868 |
| RCP10 | MCP8 | 0.355427682 |
| RCP2 | MCP8 | 0.325781703 |
| RCP4 | MCP8 | 0.494025916 |
| RCP5 | MCP8 | 0.342632771 |
| RCP6 | MCP8 | 0.365696877 |
| RCP8 | MCP8 | 0.310646147 |
| RCP1 | MCP9 | 0.582924426 |
| RCP10 | MCP9 | 0.629799366 |
| RCP2 | MCP9 | 0.48665604 |
| RCP4 | MCP9 | 0.650752306 |
| RCP5 | MCP9 | 0.612640858 |
| RCP6 | MCP9 | 0.648674071 |
| RCP7 | MCP9 | 0.4303042 |
| RCP8 | MCP9 | 0.619631648 |
| MWP19 | MWP13 | 0.462023258 |
| MWP5 | MWP13 | 0.410182089 |
| MWP6 | MWP13 | 0.443722099 |
| MWP8 | MWP13 | 0.374030918 |
| MWP5 | MWP18 | 0.406838357 |
| MWP6 | MWP18 | 0.372329593 |
| MWP6 | MWP5 | 0.804266512 |
| MWP8 | MWP5 | 0.416382849 |
| MWP8 | MWP6 | 0.463706166 |
| RCP10 | RCP1 | 0.739441514 |
| RCP2 | RCP1 | 0.610296488 |
| RCP4 | RCP1 | 0.627023101 |
| RCP5 | RCP1 | 0.690419495 |
| RCP6 | RCP1 | 0.715766132 |
| RCP7 | RCP1 | 0.459963441 |
| RCP8 | RCP1 | 0.714045584 |
| RCP2 | RCP10 | 0.561961114 |
| RCP4 | RCP10 | 0.612892985 |
| RCP5 | RCP10 | 0.731497705 |
| RCP6 | RCP10 | 0.798957586 |
| RCP7 | RCP10 | 0.515829563 |
| RCP8 | RCP10 | 0.782362044 |
| RCP4 | RCP2 | 0.539055586 |

| | | | | | |
|---|---|---|---|---|---|
| MCP9 | MCP4 | 0.720632732 | RCP5 | RCP2 | 0.610953331 |
| RCP1 | MCP4 | 0.739521921 | RCP6 | RCP2 | 0.622983515 |
| RCP10 | MCP4 | 0.829283893 | RCP7 | RCP2 | 0.539431691 |
| RCP2 | MCP4 | 0.545181036 | RCP8 | RCP2 | 0.605473459 |
| RCP4 | MCP4 | 0.617342174 | RCP5 | RCP4 | 0.596200466 |
| RCP5 | MCP4 | 0.763753295 | RCP6 | RCP4 | 0.616751611 |
| RCP6 | MCP4 | 0.806639254 | RCP7 | RCP4 | 0.370275587 |
| RCP7 | MCP4 | 0.482407481 | RCP8 | RCP4 | 0.599891067 |
| RCP8 | MCP4 | 0.766966879 | RCP6 | RCP5 | 0.752898634 |
| MCP7 | MCP5 | 0.454329908 | RCP7 | RCP5 | 0.544891954 |
| MCP8 | MCP5 | 0.352232069 | RCP8 | RCP5 | 0.709425032 |
| MCP9 | MCP5 | 0.666088462 | RCP7 | RCP6 | 0.538466036 |
| RCP1 | MCP5 | 0.655108988 | RCP8 | RCP6 | 0.756495953 |
| RCP10 | MCP5 | 0.727000415 | RCP8 | RCP7 | 0.551578343 |
| RCP2 | MCP5 | 0.546182096 | RWP15 | RWP10 | 0.743631125 |
| RCP4 | MCP5 | 0.564081609 | RWP17 | RWP10 | 0.738391817 |
| RCP5 | MCP5 | 0.708690822 | RWP6 | RWP10 | 0.897734463 |
| RCP6 | MCP5 | 0.710317016 | RWP9 | RWP10 | 0.749299645 |
| RCP7 | MCP5 | 0.495533675 | RWP17 | RWP15 | 0.791844666 |
| RCP8 | MCP5 | 0.70558399 | RWP6 | RWP15 | 0.800657392 |
| MCP8 | MCP7 | 0.637044132 | RWP9 | RWP15 | 0.835136831 |
| MCP9 | MCP7 | 0.620754838 | RWP6 | RWP17 | 0.756336451 |
| RCP10 | MCP7 | 0.407645047 | RWP9 | RWP17 | 0.798399389 |
| RCP2 | MCP7 | 0.348198891 | RWP9 | RWP6 | 0.807845056 |
| RCP1 | MCP7 | 0.373283446 | | | |

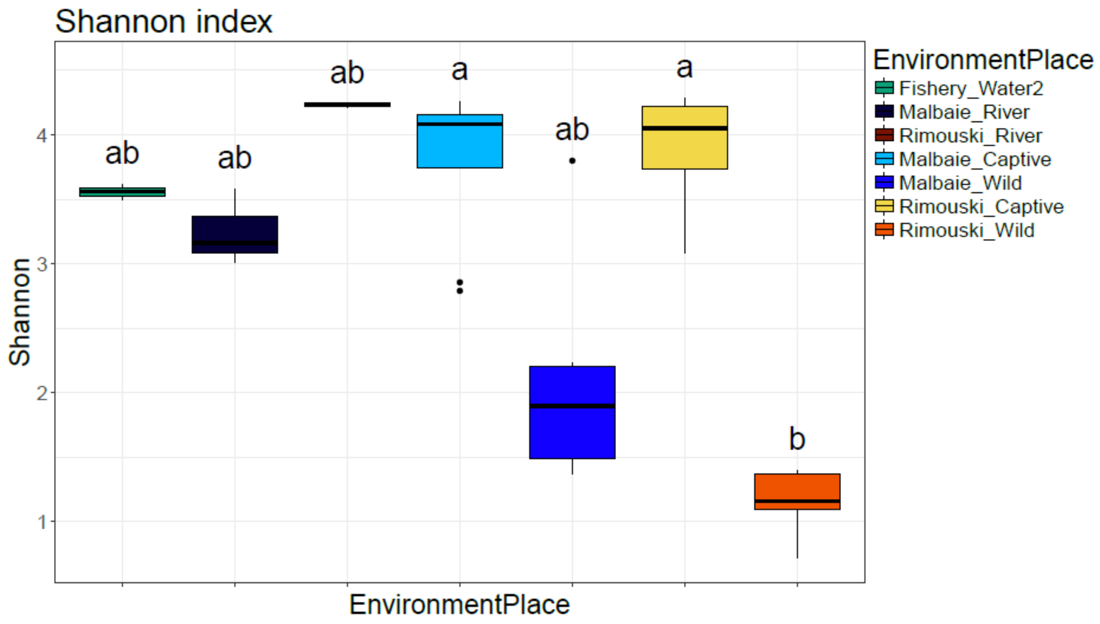


**Fig. 1** Alpha diversity of environment water and gut microbiota represented with Shannon diversity index.

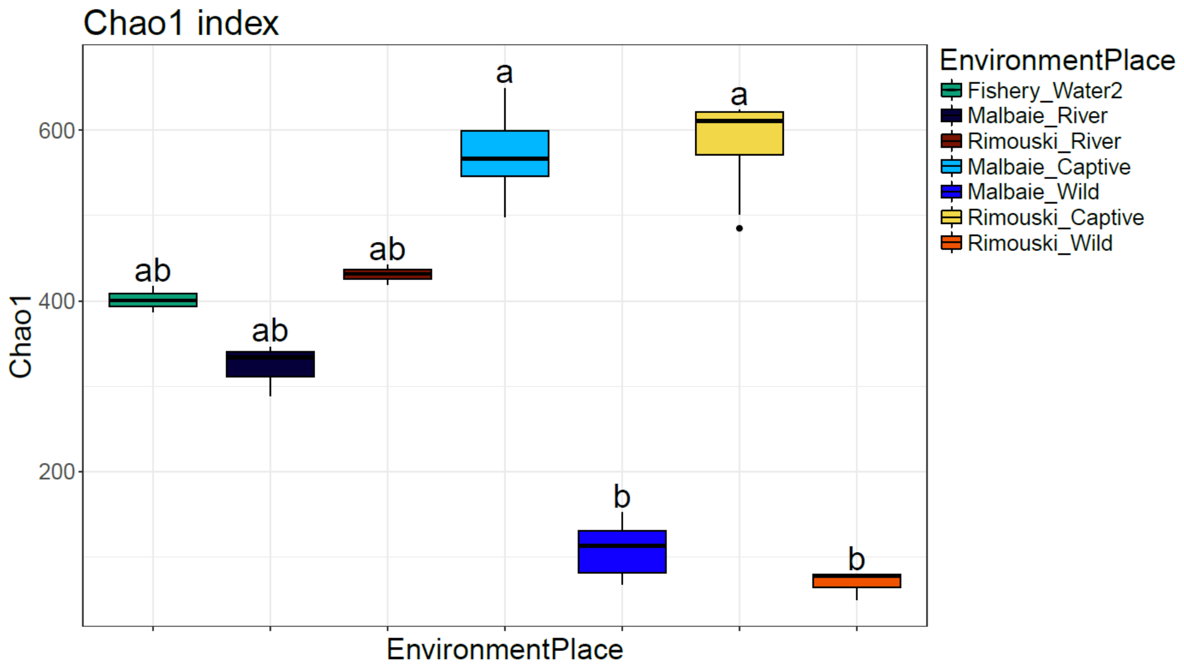


**Fig. 2** Alpha diversity of environment water and gut microbiota represented with Chao1 diversity index.

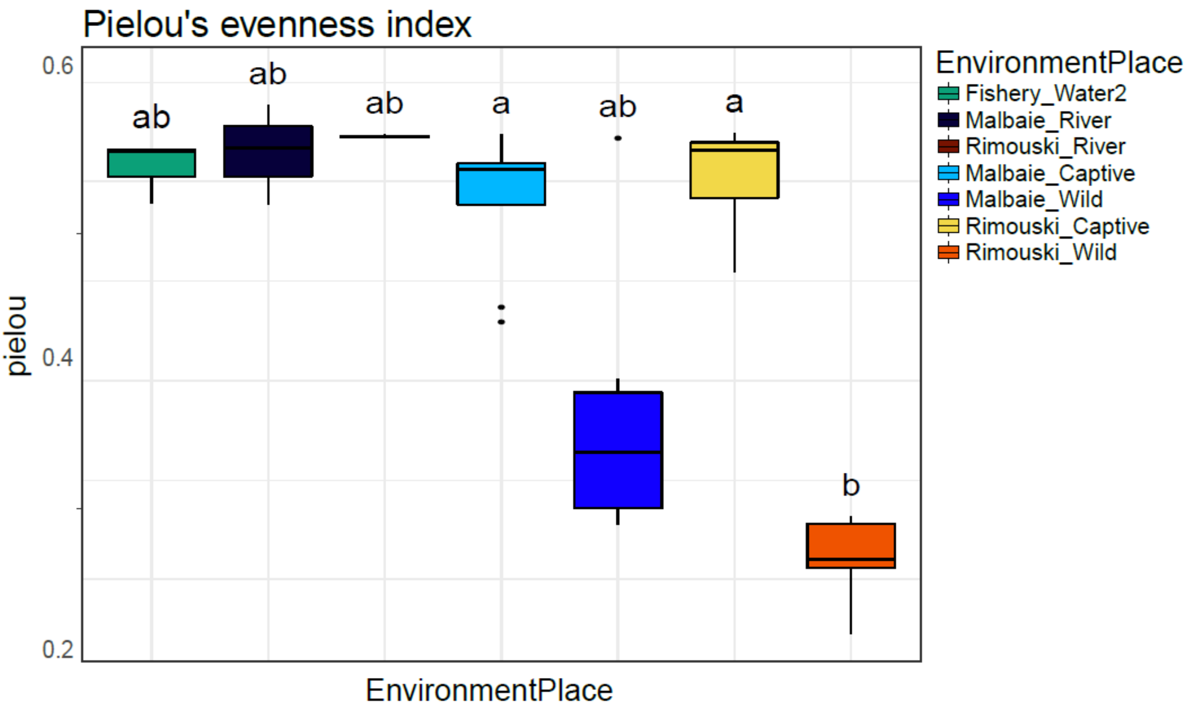


**Fig. 4** Alpha diversity of environment water and gut microbiota represented with Pielou's evenness index.

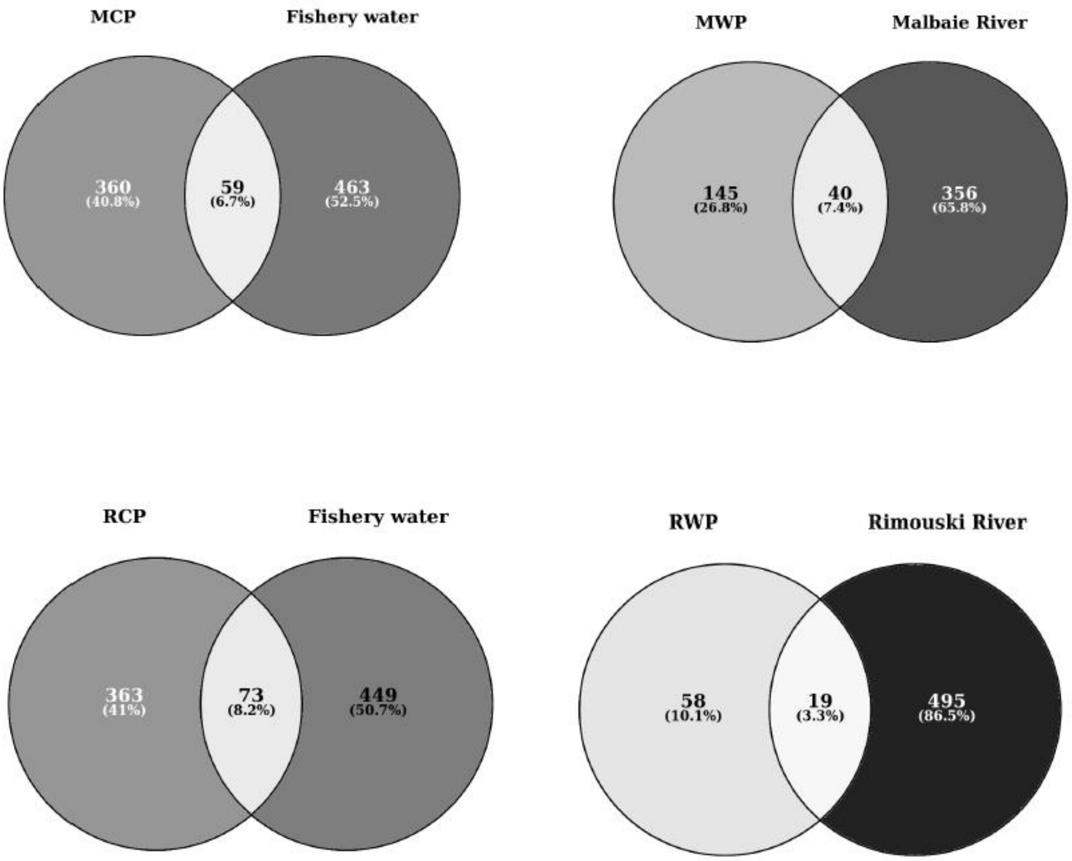


**Fig. 4** Venn diagram showing the percentage of shared OTUs between gut and environmental microbial communities. MCP; Malbaie captive parrs, MWP; Malbaie wild parrs, RCP; Rimouski captive parrs, RWP; Rimouski wild parrs.
